# Correcting a Longstanding Misconception about Social Roles and Personality: A Case Study in the Psychology of Science

**DOI:** 10.3390/bs8060057

**Published:** 2018-06-04

**Authors:** John A. Johnson

**Affiliations:** Department of Psychology, Pennsylvania State University, University Park, PA 16802, USA; j5j@psu.edu

**Keywords:** sex differences in personality, sex roles, psychology of science, motivated cognition

## Abstract

Psychologists often argue that sex roles direct different types of socializing behaviors toward males and females and that this differential treatment, in turn, leads to sex differences in personality. Widely cited in support of this thesis has been the Fels longitudinal study finding that dependency and passivity are stable from childhood to adulthood for females only and aggressiveness and sexuality for males only. The present article explains why the type of sex differences in personality stability cited by Fels researchers actually contradicts the view that sex role expectations cause these differences. The report suggests ways in which social learning theory, the dominant developmental paradigm of the 1960s, may have contributed to the misinterpretation of the Fels data and how the rise of social constructivism maintained this misinterpretation for decades. The article concludes by correcting misconceptions about biology and personality stability and by explaining why theories that incorporate biology are not only more adequate than social constructivism but also more effective in bringing about the changes in society that constructivists desire.

## 1. Introduction

“Still, a man hears what he wants to hear and disregards the rest,” goes a Paul Simon lyric [1] that encapsulates what psychologists today call motivated cognition [2]. The study of motivated cognition dates back to the “New Look” psychology of the 1950s. The New Look movement was initiated by Jerome Bruner [3], Hadley Cantril [4], and Leo Postman [5]—all students of Gordon Allport [6]. New Look psychology reacted against the then-prevalent view of the perceiver as “a passive recording instrument” [7] (p. 33). The central thesis of New Look psychology offered an alternative view: “The organism in perception is in one way or another in a state of expectancy about the environment” [5] (p. 206). The New Look reconceptualized perception as a top-down process in which “the percept is significantly modified by expectations, values, emotions, needs, and other factors that are endogenous to the perceiver” [8] (p. 201).

Philosopher of science N.R. Hanson [9] recognized that the thesis of New Look psychology applied to the cognitive activities of scientists as well as non-scientists. Hanson coined the catchphrase, “perception is theory-laden” to denote how factors endogenous to a scientist shape the way that scientist sees the world. This idea was developed further by historian and philosopher of science Thomas Kuhn [10], whose work legitimized psychological studies of science [11].

The current article describes a case study in the psychology of science. Specifically, this report suggests how motivated cognition apparently caused researchers in the Fels Longitudinal Study [12] to draw conclusions about sex-differentiated personality stability that were precisely opposite of what the data indicated. Furthermore, prevailing social constructivist paradigms in developmental psychology continued to blind psychologists to this misinterpretation for decades following the publication of Kagan and Moss’s book [12]. This report concludes by discussing how motives are better served by striving for truths about preexisting realities rather than by attempting to construct support for one’s favored view.

## 2. The Famous but Incorrect Fels Interpretation of Sex Differences in Personality Stability

### 2.1. Historical Significance of the Fels Study

In his own renowned longitudinal study of personality, *Lives Through Time*, Jack Block [13] identified research undertaken at the Fels Research Institute as one of three major, comprehensive, longitudinal studies of personality development. After describing the Fels study and noting its limitations, Block concluded his review by declaring that psychologists “can clearly recognize the great and signal contribution of the Kagan and Moss study” [13] (p. 7). Developmental psychology textbook author David Shaffer likewise described the Fels study as “one of the most famous longitudinal projects in the history of developmental psychology” [14] (p. 28).

### 2.2. The Fels Interpretation of Sex Differences in Personality Stability

One of the more frequently cited findings from the Fels study is that sex role-prescribed personality traits (aggressiveness and sexual initiative in males, passivity and dependence in females) show more stability from childhood (ages 6–10) to adulthood (ages 19–29) than non-sex role-prescribed traits. Trait stability was defined in the study as the Pearson correlation between measurements taken during childhood and later during adulthood. Kagan and Moss also report significant correlations for overall sex-typed activities for both males and females across the same time period. They interpret this differential stability as a consequence of socializing agents who reinforce behavior that is consistent with society’s prescribed gender roles:

“Passive and dependent behavior are subjected to consistent cultural disapproval for men but not for women. … It is not surprising, therefore, that childhood passivity and dependency were related to adult passive and dependent behavior for women, but not for men” [12] (p. 268) and “the individual’s desire to mold his overt behavior in concordance with the culture’s definition of sex-appropriate responses is a major determinant of the patterns of continuity and discontinuity in his development”.

[12] (p. 269)

### 2.3. Why the Fels Conclusion Is a Misinterpretation of the Data

Significant correlations between personality measurements over time are actually the opposite of what one would expect if social expectations were molding personality. Consider the following facts. Sex role stereotypes, whatever their accuracy, are cross-culturally pervasive: women are expected to be expressive and nurturing, and men instrumental and agentic [15,16]. If these pervasive social expectations about sex-appropriate behavior were potent influences on a child’s developing personality, these expectations would, over time, restrict trait levels toward the portion of a personality continuum deemed appropriate by society. To illustrate this idea with aggressiveness, one of the traits assessed in the Fels study, boys (but not girls) initially rated low in aggressiveness would be seen as more aggressive over time because society expects boys (but not girls) to be aggressive. On the other hand, girls initially rated high in aggressiveness would be seen as less aggressive over time.

**Figure 1:**
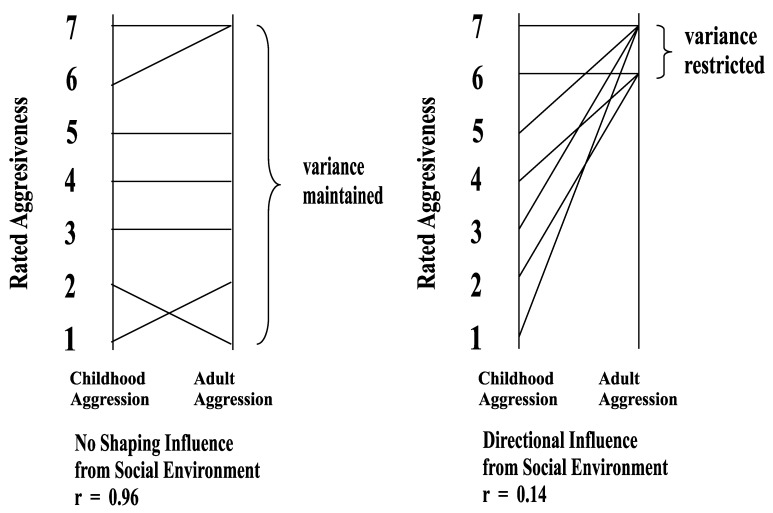
Illustration of how hypothetical social environmental influences would restrict variance, leading to personality instability.

In short, successful socialization into sex roles (e.g., shaping males to be aggressive or females to be dependent) would restrict the range of personality differences within each sex over time. How would this affect correlation coefficients between trait measurements gathered over time? When a set of personality scores is limited to a narrow range of a scale, this reduced variance imposes an upper limit on the correlation between those scores and other measurements [17] (pp. 186–187). If all individuals reached exactly the same level on a personality dimension, the correlation between this set of scores and scores gathered earlier would be zero. In a nutshell, the more successful the socialization, the less stability we should find in individual differences. This point is illustrated graphically in Figure 1.

The stability that Kagan and Moss observed for aggressiveness and sexual initiative (for boys) and passivity and dependency (for girls) actually contradicts the hypothesis that these traits are shaped by the social expectations. Stability indicates that boys who show sexual initiative continue to show sexual initiative as men, but also indicates that boys who show little sexual initiative tend to show little sexual initiative as adults, despite society’s expectations for males to show sexual initiative. Likewise, stability indicates not only that passive girls become passive women but also that assertive girls become assertive women, despite norms against assertiveness in women.

The argument here is not that the significant correlations for gender-related personality traits over time rule out the possibility of socializing influences. Only a correlation of 1.00 would indicate complete imperviousness to such influences. The point is that Kagan and Moss should not have interpreted the relatively larger magnitude of stereotypic masculine and feminine trait correlations (compared to correlations for non-gender-related traits) in the Fels study as evidence for the power of socializing influences.

There is another way to demonstrate the fallacy in Kagan and Moss’s reasoning without invoking arguments about statistical restriction of range. Consider that ratings of any trait can be translated as an inverse score on the trait’s opposite. For example, on a 1–7 scale for rating aggressiveness, someone who is rated a “7” on this aggressiveness scale could be identically characterized as a “1” on a non-aggressiveness scale. A “6” on aggressiveness is equivalent to a “2” on non-aggressiveness, and so forth. Correlations between aggressiveness scores gathered at two points in time would have exactly the same absolute magnitude as non-aggressiveness scores gathered at two points in time. Whereas Kagan and Moss (1962) report that aggressiveness is a relatively stable trait for males, one could just as easily score their scales in reverse and conclude that non-aggressiveness is a relatively stable trait for males. Clearly, society’s expectations for males to be aggressive cannot be causing both that stability of aggressiveness and the stability of non-aggressiveness. Temporal stability coefficients simply do not demonstrate socializing influences.

## 3. Why Did Kagan and Moss Get It Wrong?

### Theory-Laden Confusion of the Two Meanings of Stability

Some 56 years ago, members of the Fels Research Institute team were poring over and attempting to make sense out of matrices of correlation coefficients from their longitudinal study. Someone must have noticed an apparent pattern between the size of the coefficients and whether the trait was prescribed by the male or female sex role, because this is the finding that Kagan and Moss reported in their book [12]. But by what reasoning could the researchers have moved from noticing that the correlations for male-role-consistent traits and female-role-consistent traits were larger than sex-role-neutral traits, to concluding that the apparent sex-specific personality stability was caused by society’s sex-role expectations?

Reconstructing events from over five decades ago would likely be difficult even for the Fels researchers themselves. Pending a better explanation, I suggest that the Fels researchers may have slipped between two different meanings of personality stability. Personality stability can refer either to stable mean scores on a personality measure over time or to stable rank order on a personality measure over time [13,18]. The Fels study documented only the latter kind of stability, represented by significant correlations between personality measurements made at two points in time. As the Fels researchers put into words the patterns they perceived, they may have said or written something like “Aggressiveness and sexual initiative are stable for males and passivity and dependency are stable for females.” If the word “stable” was misconstrued to indicate mean stability, this would have led to the misinterpretation that high mean levels of male aggressiveness and sexual initiative and high mean levels of female passivity and dependency are stable over time.

Scientific paradigms create mind-sets that shape the perceptions and interpretations of scientists. Perhaps Kagan and Moss’s misinterpretation of their data was driven by the perception-shaping influence of the prevailing developmental paradigm of that time—social learning theory [19,20,21]. Social learning theory assumes that social expectations shape personality. The researchers were so intent on making their findings consistent with social learning theory that rank-order stability was apparently misperceived as mean stability for each sex.

## 4. The Promulgation of the Erroneous Fels Conclusion

### 4.1. The Staying Power of the Erroneous Fels Conclusion

How widespread did the Fels misinterpretation of gender-specific personality stability become? One indication of how thoroughly an idea has penetrated a field is the degree to which the idea appears in basic textbooks. For decades after the book’s publication, the Fels interpretation of sex differences in personality was cited in developmental textbooks [22,23,24,25,26]. Although direct citations may have decreased more recently, Kagan and Moss’s (1962) book is still recognized as a cornerstone of modern social role theory, which assumes that social expectations are responsible for the stability of sex-typed personality traits [27].

### 4.2. Failure of the Fels Finding to Replicate

The Fels misinterpretation has persisted despite the fact that other research has not replicated the finding of gender-specific personality stability. Parke and Slaby [28], for example, report appreciable stability in individual differences in aggression for girls. Block [13] found the item “Behaves in dependent fashion” to correlate r = 0.50 (corrected for unreliability) for boys, and the item “Expresses hostile feelings directly” to correlate r = 0.32 for girls across the time. For girls, “Tends to construe or define many different contexts in sexual terms; eroticizes situations” correlated r = 0.27 from adolescence to adulthood, and “Becomes emotionally involved with members of the opposite sex” correlated r = 0.60 across the same time period. These statistically significant coefficients of stability for gender-inappropriate traits suggest that Kagan and Moss’s findings lack generalizability and do not support their conclusion that social expectations make some personality traits more stable for males and others for females. How, then, could their misinterpretation have persisted?

### 4.3. Desire for Freedom as a Motive for Continued Acceptance of the Kagan-Moss Misinterpretation

When scientists persist in holding incorrect views, unsupported or even contradicted by evidence, chances are that an emotional commitment [29] to a philosophical [30] or political [31,32] position may be involved. One emotional commitment that may explain the continued acceptance of Kagan and Moss’s misinterpretation of their data is an attachment to the concept of free will or self-determination over the idea that human behavior is determined by laws of nature outside of our control. In the words of Rhoda Unger, the question is whether “reality constructs the person” or “the person constructs reality” [33] (p. 17). At issue are questions about whether the mind is a product of physical matter, subject to the laws of biology, chemistry, and physics, or whether human beings can somehow transcend their biology and physical environment to become self-determining through autonomous thinking and free will [30].

The notion that people possess enduring, stable personality traits of any sort is an anathema to many psychologists (both professional and lay) who value individual freedom and self-determination [34]. To possess stable personality traits is to be stuck into what Walter Mischel called “fixed slots” [35] (p. 740) or “fixed positions” [36] (p. 351). For Mischel and others who value freedom and self-determination, the real reason for rejecting stable, enduring personality traits is not lack of empirical evidence for stability but because the concept of personality stability is perceived to undermine freedom [34,37].

However, one cannot argue scientifically against stable personality traits by saying, “I don’t like the idea of stable traits because it contradicts the value I place on freedom.” So, initially, trait deniers attempted to marshal evidence that stable personality traits do not exist, that behavior depends upon the situation more than on traits. Thus, the person-situation debate in psychology [38] raged on for two decades despite the logical impossibility of demonstrating that situations or personality traits are stronger determinants of behavior. Asking whether situations or personality traits contribute more toward behavior is like asking whether the properties of a solvent or crystalline substances contribute more toward dissolving behavior in chemistry [39,40]. Chemists long ago understood that such a question would be pointless and that the appropriate research question is, “What is it about the structure of substances and liquids such that in some cases dissolving occurs, and in other cases, it does not?” [40] (p. 251). The answer lies in the structure or crystalline materials and solvents. In contrast, “situationists” avoided questions about the stable personality structures, instead trying to demonstrate that environmental situations are always stronger determinants of behavior than personality traits.

“Situationism” was comfortably consistent with social learning theory of the early 1960s. Rooted in behaviorism, early social learning theories stressed environmental reinforcers as determinants of a person’s behavior [27,33]. According to social learning theory, parents reward their sons and daughters for sex-role-appropriate behavior and punish inappropriate behavior. As children develop, their peers continue to reinforce masculine behavior for boys and feminine behavior for girls. This reinforcement persists throughout adulthood. Expected behaviors for men and women have been consistent over across cultures for decades, despite changes in sex roles and attitudes toward them in modern, western cultures [15]. Social learning theory’s assumption that social expectations for men and women cause sex differences in personality is what led Kagan and Moss to misinterpret their correlations as evidence that masculine traits are more stable for males and feminine traits are more stable for females. Psychologists who wanted women and men to be free from gender stereotyping accepted this misinterpretation because they believed that gender equality could be achieved by simply changing attitudes about sex roles in society.

But not all psychologists who valued freedom saw situationism as a way out of the perceived prison of sex-typed personality traits. Among these psychologists was Walter Mischel himself. Mischel expressed disconcertedness about his publications being cited approvingly by situationists, and he denied being a situationist [35,36]. Mischel realized that attributing behavior to situations was just as deterministic as attributing behavior to personality traits. Consequently, Mischel’s versions of social learning theory became less behavioristic in wording and more cognitive over time. His original view of the development of sex differences leaned heavily on the traditional behavioristic concept of environmental reinforcement [40]. Mischel later added cognitive variables to his theory and renamed it cognitive social learning theory [41]. Eventually, his work with Nancy Cantor on cognitive prototypes in person perception [42] contributed to the cognitive-schema approach in personality, social psychology, and gender [43,44]. As Mischel’s social learning theory became more cognitive, it abandoned the behaviorist assumption that fixed realities create people and adopted the position that people create reality. Cognitive social learning theory holds that if there is consistency of behavior, it is not due to the environmental situation or fixed personality traits but because of active cognitive processes that lead to free choices to be consistent.

Whereas Walter Mischel has been dedicated to arguing for freedom and flexibility for everyone, feminist psychologists have been particularly concerned about women’s freedom to become whomever they choose to be. They have objected to the notion that women have fixed, feminine traits that limit their freedom. Feminist psychologists have been particularly dismissive of claims that women have traits that make them inferior to men, claims that have been used to justify paying women less than men for the same work and excluding women from high-paying jobs and positions of leadership and power. In the 1970s, when feminist psychologists began their own research that compared men and women, they expected to disconfirm stereotypic sex differences in personality traits and abilities, thereby increasing women’s chances for equal opportunity in society [32].

However, by the mid-1990s, meta-analyses of research on sex differences demonstrated that moderate sex differences exist for some personality traits, that research finding these differences is more consistent than inconsistent, that these results are not a function of artifacts, and that the specific findings support the stereotype that women tend to have higher levels of empathy and nurturance while men tend to have higher levels of aggressiveness and toughness [32].

Despite the fact that people have a more favorable overall view of women than men [32], some feminists were displeased by the confirmation of stereotypic, sex-related personality traits. Because high-status role in society were thought to require aggressiveness and toughness, these feminists did not want to believe that men are inherently tougher or more aggressive than women. Some feminists therefore began arguing that personality traits themselves are mere constructions about reality rather than objective characteristics of reality. In the words of Rhoda Unger, the person constructs reality rather than reality constructs the person [33].

The position that people construct reality, often called constructivism, dismisses objective, physical reality as a determinant of human behavior. Constructivists claim that we determine our own behavior by the way in which we think about reality. Constructivism that involves conversations with other people is called social constructivism. According to social constructivists, social interaction creates meanings, and these jointly-constructed meanings govern subsequent social interaction [45]. From the perspective of social constructivism, traits such as empathy, nurturance, aggression, and toughness and differences between men and women on these traits exist only in our conversations, not as objective facts about an independent reality. Social constructivists believe that freedom from the constraints of personality language can be achieved simply by changing the conversation.

### 4.4. Social Constructivists’ Political Arguments against Biology

Social constructivists regard conversations about biology as especially harmful to freedom. Constructivists often assume that describing personality traits as biologically-based implies that these traits are “stable, universal, and immutable” whereas speaking of traits as socially constructed means that the traits “are more variable and easily changed … (Consequently) neither social learning, cognitive developmental, or schema approaches have approached the consideration of biological influences on gender-role development in systematic ways” [46] (p. 965). According to Jeanne Marecek, constructivists do not necessarily deny biological differences between the sexes, “(b)ut they do deny that such differences have a single, fixed meaning and salience whether from one culture to another, one historical period to another, one social group to another, or even from time to time in an individual’s experience” [45] (p. 162). As far as constructivists are concerned, biology is “just another way of knowing, and, moreover, contrived mostly by European and American white males” [47] (p. 45).

For individuals who want to increase the economic status and political power of women, social constructivism is a hopeful message that reformers want to believe because economic and political equality can be achieved simply by changing the way we converse about women and men. In contrast, the idea that immutable biological factors underlie status and power seems to be a message of despair; it is a message that reformers do not want to believe. Social constructivist assumptions are bolstered by American ideology, which holds that people are born equal and that anyone can achieve the American dream in this land of opportunity [48,49]. When the desire for freedom and equality causes people to adopt social constructivism, this is an example of motivated cognition.

Psychologists have generally adhered to the traditional scientific assumption that a natural reality existed before human beings evolved on this planet and that this preexisting reality creates human beings rather than the other way around. Although psychologists might be sympathetic to the view that people construct models or representations of reality with language, the standard scientific view is that people do not construct reality itself. Furthermore, the usefulness of a model of reality depends on its verisimilitude, by its goodness-of-fit with reality as it actually is. To think that persons literally construct reality with their thoughts and conversations would strike most psychologists as wishful thinking or even magical thinking.

Wishful thinking that underlies an argument is a logical error sometimes referred to as the moralistic fallacy [50,51]. The moralistic fallacy (“X ought to be; therefore X is”) is the converse of the naturalistic fallacy (“X is; therefore X ought to be”). In the present context, social constructivists who wish people to be free from limiting, gender-related personality traits commit the moralistic fallacy when they propose arguments such as “Men and women ought to be equal. Therefore, women are just as strong as men and men are just as empathetic as women [52]”.

This is not to say that only social constructivists have personal agendas that lead them into logical fallacies. Biologically oriented researchers have also demonstrated motivated cognition and fallacious thinking. Darwin himself thought that men were inherently intellectually superior to women, and it is now common knowledge that arguments about what is biologically natural have been used to justify racism and sexism for at least 100 years after Darwin [53]. But motivated cognition can also be seen in biologically oriented researchers with good rather than dark intentions. Consider the following admission by Judith Harris [54], who wrote a book arguing that parents have little influence on the way their children turn out: “One of Harris’s ‘primary motivations for writing the book,’ she says in an e-mail, was ‘to lighten the burden of guilt and blame placed on the parents of “problem” children’” [55] (p. 59). Although parents have thanked her for this, some psychologists have accused her of rationalizing the neglect and mistreatment of children. Among them was the same Jerome Kagan who misinterpreted correlations in the Fels Institute study. Concerning Judith Harris’s motives, he said, “I am embarrassed for psychology” [55] (p. 54).

Some persons believe that an inquiry into the personal agendas underlying research automatically constitutes an inappropriate, ad hominem (or ad feminam) attack on the researcher. Others [56,57,58,59,60,61,62,63] believe that studying the psychological processes that lead to scientific knowledge claims can further increase our knowledge of a subject domain.

In their chapter, “Scientists are People,” McCain and Segal [64] discuss the various motives that underlie research. That research is motivated by the personal concerns of researchers seems to be inevitable, but this is not necessarily a lamentable state of affairs. From the social constructivist viewpoint, personal motivations are problematic only when they fail to help realize the goal of increased freedom. From a naturalistic viewpoint, personal motivations are troublesome only when they lead us away from truth. The final section of this article suggests that the goal of increased freedom can be achieved more effectively by accepting rather than denying biological truths.

## 5. Conclusions: The Truth about Biological Stability and Change

Times have changed over the last 55 years. At the time of the Fels research (the early 1960s), psychology was still dominated by behavioristic social learning theory, which claimed that the mind is a blank slate at birth and that the social environment creates personality. This view was so strong that it led Kagan and Moss to interpret their data on gender-related personality traits backwards, claiming that social expectations caused males to acquire stable masculine traits and females to acquire stable feminine traits. Feminist psychologists in the 1970s hoped that, as attitudes about sex roles changed and society began treating men and women more equally, research would show no significant personality differences between men and women. By the 1990s, when research clearly supported the reality of some stereotypic sex differences in personality, social constructivists challenged the notion of fixed, biological realities and argued that we are free to create any realities we desire by changing our thinking and conversations.

By the end of the millennium, the view of mind as a blank slate had been thoroughly disconfirmed by temperament and behavior genetics research [65,66]. Research has described both the evolution of genetically based personality differences between the sexes [67,68] and also genetic components of individual differences in masculinity and femininity within each sex [69,70]. Kagan himself eventually studied biologically based temperament [71,72,73]. Several large-scale studies strongly replicated sex differences in personality within the now predominant description of personality structure, the Five-Factor Model [74,75,76,77]. (Note, however, that not all well-established sex differences in personality traits fall within the Five-Factor Model. For example, interests and values incline men and women disproportionately toward higher- and lower-paying occupations [78].)

Surprising to those who believe that society shapes personality, actual sex differences in personality have been found to be stronger in progressive Western societies than in traditional societies—just the opposite of constructivists’ predictions that liberal social attitudes about sex roles would decrease sex differences in personality. Today, we also now have much more information about personality change and stability (both rank-order and mean-level) over the lifespan from large meta-analyses [79,80]. The findings from these studies are many and complex, but two findings are that rank-order personality stability generally increases with age, and mean personality levels slowly increase or decrease over the lifespan, depending on the trait. Importantly, in neither case is stability not depend on whether the trait is gendered. 

Researchers who reject biological explanations because they are perceived to interfere with the political goals of addressing gender inequality are making two serious mistakes [81]. First, the assumption that biologically based traits are “stable, universal, and immutable” [46] (p. 965) simply is not true. Biology entails both stability and change. A living organism must possess at least some stability; otherwise, it would not be recognized as the same organism from one moment to the next. On the other hand, individual organisms undergo both predictable and unpredictable changes over the life cycle, and species evolve over longer periods of time. To take a naturalistic (as opposed to a supernatural or dualistic) view of learning, a biologist would say that every bit of social-role learning involves an objective, real change in the brain of the learner. Therefore—contrary to the claims of feminist constructivists—studying the biological substrates of sex differences does not automatically translate into a defense of the status quo.

Second, stating that something is true because one wants it to be true (the “moralistic fallacy”) hardly seems like an effective way to make the world a better place for anyone. Without denying that science can be misused or abused, one can safely say that the basic naturalistic assumption of science (that nature behaves a certain way regardless of how we want it to behave) has given us the power to radically change the way we live. Reality does create persons. If we want to change our lives for the better, acknowledging the real basis of gender-related personality traits will take us there more effectively than denying that we are subject to the laws of human nature, whatever they may be.
